# The foundation for the microbiology laboratory’s essential role in diagnostic stewardship: an ASM Laboratory Practices Subcommittee report

**DOI:** 10.1128/jcm.00960-24

**Published:** 2024-09-30

**Authors:** Rebekah E. Dumm, Elizabeth M. Marlowe, Logan Patterson, Paige M. K. Larkin, Rosemary C. She, Laura M. Filkins

**Affiliations:** 1 Department of Pathology and Immunology, Washington University School of Medicine, St. Louis, Missouri, USA; 2 Quest Diagnostics, San Juan Capistrano, California, USA; 3 University of North Carolina, Chapel Hill, North Carolina, USA; 4 American Society for Microbiology, Washington, DC, USA; 5 Department of Pathology, City of Hope National Medical Center, Duarte, California, USA; 6 Department of Pathology, University of Texas Southwestern Medical Center, Dallas, Texas, USA; Vanderbilt University Medical Center, Nashville, Tennessee, USA

**Keywords:** diagnostic stewardship, regulatory compliance, laboratory accreditation, infectious disease diagnostics

## Abstract

Diagnostic stewardship (DxS) has gained traction in recent years as a cross-disciplinary method to improve the quality of patient care while appropriately managing resources within the healthcare system. Clinical microbiology laboratorians have been highly engaged in DxS efforts to guide best practices with conventional microbiology tests and more recently with molecular infectious disease diagnostics. Laboratories can experience resistance to their role in DxS, especially when the clinical benefits, motivations for interventions, and underlying regulatory requirements are not clearly conveyed to stakeholders. Clinical laboratories must not only ensure ethical practices but also meet obligatory requirements to steward tests responsibly. In this review, we aim to support clinical microbiology laboratorians by providing the background and resources that demonstrate the laboratory’s essential role in DxS. The heart of this review is to collate regulatory and accreditation requirements that, in essence, mandate DxS practices as a long-standing, core element of high-quality laboratory testing to deliver the best possible patient care. While examples of the clinical impact of DxS are plentiful in the literature, here, we focus on the operational and regulatory justification for the laboratory’s role in stewardship activities.

## INTRODUCTION

The goal of diagnostic stewardship (DxS) in laboratory medicine is to improve diagnostic accuracy and optimize patient treatment (1). The pillars of a DxS program can be described using the four Rs, which ensure (i) the right tests are ordered, (ii) for the right patient, (iii) at the right time, and (iv) for the right clinical management (2). Additional goals of DxS are to promote antimicrobial stewardship (AS) and optimize the utilization of institutional resources. This paper will focus on DxS for infectious diseases in the clinical microbiology laboratory.

The theory and practice of DxS have evolved with time, but DxS has been a core element of clinical laboratory quality and patient safety since before Clinical Laboratory Improvement Amendments (CLIA) ’88 regulations were enacted in the United States. Originally DxS activities focused on optimizing specimen collection, analytical processing, and accurate reporting of testing results. Interventions now also target improved patient outcomes and antimicrobial utilization (3). To accomplish these goals, effective stewardship teams are often made up of multidisciplinary stakeholders and focus on quality improvement. DxS can be implemented anywhere along the testing journey from ordering to analytical processing and testing to reporting of results. Given that range, many interventions benefit from laboratory involvement and often from laboratory leadership as part of cross-disciplinary DxS teams. The goal of DxS is not to reduce testing, but rather to guide value-based targeted interventions to improve the continuum of care (i.e., screening, diagnosis, monitoring, and treatment) (4). To that end, many DxS practices are also a component of AS programs, where appropriate diagnostic testing can support the goal of appropriate antimicrobial utilization. Experts from the laboratory, infectious diseases, and pharmacy have all contributed to guidance documents, underscoring the importance of choosing appropriate testing (5).

DxS can face scrutiny when its objectives are inaccurately equated with supplanting a provider’s autonomy to select a test or when stakeholder support for a DxS intervention is lacking. In contrast, a comprehensive DxS program is one that supports optimal testing in all phases, not just test selection, and in which clinical providers collaborate with the laboratory and other stakeholders to mutually select interventions aimed at improving patient care. The objective of this article is to outline the operational and regulatory requirements of laboratories in the United States to carry out DxS and present evidence for its potential clinical impact. This document can serve as a reference with centralized information to aid clinical laboratories in communicating and, when necessary, justifying their role in driving DxS collaborations with key stakeholders.

## THE BENEFITS OF DIAGNOSTIC STEWARDSHIP

DxS encapsulates many strategies to improve patient care by encouraging the appropriate utilization and interpretation of diagnostic testing (Table 1). As laboratory testing continues to increase in both volume and complexity, promoting the appropriate utilization of tests requires increasingly multidisciplinary teams and continuous effort (6–9). The most sustainable improvements often employ multilayered strategies, combining systems-level approaches with behavioral strategies (10, 11), which the laboratory is well-suited to spearhead (12, 13). Common strategies focus on the overutilization of high-volume tests, such as blood cultures, urine cultures, and testing for *Clostridioides (Clostridium) difficile* infections (1). Newer technologies, such as multiplex molecular panels and sequencing methods for the diagnosis of infectious diseases, also benefit from DxS efforts due to challenging interpretations and higher costs with limited reimbursement.

**TABLE 1:** Examples of diagnostic stewardship goals and potential benefits to patient care^*a*^

DxS goals	Example interventions
Optimize specimen collection and transport to reduce recollections and delayed results and improve result quality	Provide education and electronic tools to guide proper specimen labeling, collection volume, collection device, and transport (14, 15)
Encourage clinically accurate result interpretation to guide optimal care and avoid harm	Employ HIV, hepatitis B, and hepatitis C diagnostic algorithms Reflex testing and/or requiring clinical indications for *C. difficile* diagnosis (16, 17) Clinical indications for blood cultures (18) Laboratory or infectious disease consultation before metagenomic next-generation sequencing
Support optimal antimicrobial usage	Optimizing clinical indications for, collection of, and result reporting from urine cultures (19)
Improve healthcare resource utilization	Optimizing use and AS follow-up on results from rapid molecular tests (20) Avoid lower respiratory cultures testing for low-yield clinical scenarios (21)
Improve healthcare system metrics	Optimize urine and blood specimen collection to avoid detection of clinically insignificant organisms that are categorized as healthcare-associated infections (22)
Avoid unnecessary patient bills	Employ clinical algorithms to guide rapid respiratory test selection and reduce overuse of broad panels (23)

^*a*^ DxS toward many of the examples shown below have multiple benefits and are not limited to the category shown.

Interpreting the results of an inappropriately ordered test or suboptimally collected specimen can contribute to diagnostic error by both over-diagnosis and missed diagnosis (1). For example, overutilization of blood cultures and suboptimal blood culture specimen collection methods can lead to increased detection of contaminating microorganisms, resulting in increased length of stay, unnecessary antimicrobial treatment, and additional downstream testing (18). Many DxS efforts address appropriate collection practices, proper indications, and guidance for interpreting contaminated cultures (24). Also frequently misdiagnosed, asymptomatic bacteriuria or a positive urine culture in a patient without genitourinary symptoms causes significant antibiotic prescribing in both inpatient and outpatient settings (25). Efforts to reduce inappropriate treatment of asymptomatic bacteriuria have largely focused on reducing overutilization of urine testing, spanning system-based reflex-based order sets to educational interventions (19).

Other DxS approaches share the goal of reducing uncertainty in interpreting the subsequent test result(s) to improve diagnostic accuracy but instead focus on optimizing which analytic method(s) to apply. For example, testing algorithms for the diagnosis of bloodborne viral infections due to HIV, hepatitis B virus, and hepatitis C virus apply screening and confirmatory tests in a specific sequence to optimize both diagnostic sensitivity and specificity. Meanwhile, detection of *C. difficile* in hospitalized patients can suffer from low clinical specificity due to asymptomatic colonization, resulting in over-diagnosis of *C. difficile* infections if not accounted for in the ordering and performance of testing (16). Reflex testing with a second method, computerized clinical decision support, and educational efforts around appropriate test indications contribute to a multifaceted approach that curbs erroneous *C. difficile* infection diagnoses (16, 17).

Since inappropriate testing can lead to inappropriate antimicrobial prescribing, DxS is considered a preceding element of AS. Retrospective audit and feedback have long been pillars of AS programs. When combined with a proactive emphasis on selective and cascade reporting of antimicrobial agents, DxS and AS combine to guide prescribing habits and benefit individual patient care and public health more broadly (26, 27).

A secondary goal and benefit of DxS is to improve the efficiency of healthcare and the allocation of finite resources. While measuring the contribution of laboratory testing to the overall efficiency of clinical care can be challenging, improved efficiency has been demonstrated through faster triage in the Emergency Department or time to removing patients from isolation rooms (28, 29). Laboratory involvement in technical workflows and institutional ordering guidelines can also aid hospitals in meeting goals around reductions in healthcare-acquired infection rates (22), and thoughtful DxS interventions can promote high-yield testing strategies and free up resources by reducing unnecessary testing (30). In the post-COVID era, staffing shortages have reached an all-time high (31), necessitating the conservation of laboratory resources. It is also important to note that since DxS efforts focus on both overutilization and underutilization of diagnostic testing, certain interventions can increase the costs and effort to the laboratory, which is much more challenging to measure (32).

## REGULATORY REQUIREMENTS FOR DIAGNOSTIC STEWARDSHIP

Laboratories in the United States are bound by the requirements legislated by CLIA ’88 and by deemed accreditation agencies, including The Joint Commission, the College of American Pathologists (CAP), and others. To maintain compliance, the laboratory must coordinate a program to ensure quality across the pre-analytic, analytic, and post-analytic phases of testing. To accomplish this, DxS is not just beneficial—it is required.

The following are examples of commonly encountered questions or scenarios in which laboratories routinely participate in DxS activities to ensure high-quality testing by complying with federal regulation and laboratory accreditation requirements. Key regulations and example accreditation requirements are also summarized in Table 2. Laboratories may consider these examples and how the associated evidence of DxS compliance could similarly apply to high-priority DxS scenarios at their institution or to support the basis for formalizing a multidisciplinary institutional DxS program.

**TABLE 2:** Regulatory and accreditation compliance achieved by diagnostic stewardship activities^*a*^

Stage of testing	Stewardship activity	Highlighted regulatory or accreditation requirements
Test evaluation	Validating and verifying test performance is appropriate for the population served	**CLIA §493.1253** and **CAP COM.40640:** verification of test performance **CAP MOL.31015:** specimen types for molecular assay validation studies
	Selecting appropriate reference services to ensure high-quality, clinically indicated testing to meet the needs of the population served and meet federal regulations	**CLIA §493.1445:** lab director responsibilities **CAP GEN.41350:** referral lab selection **JC LD.04.03.09 EP1** and **EP10:** referral lab selection
Pre-analytic	Ensuring sample types and clinical indications meet FDA approval or laboratory validation	**JC DC.02.01.01 EP 1:** lab procedure requirements **CAP COM.40640:** LDT clinical performance validation
	Upholding quality of testing by ensuring proper specimen submission, handling, and referral	**CLIA §493.1240:** evaluating and monitoring pre-analytic specimen quality **CLIA §493.1242:** policies and procedures for pre-analytic specimen steps
	Clinically available and user-friendly procedures to guide appropriate testing	**JC QSA.15.01.01 EP2:** molecular testing policy requirements
	Consultation to guide appropriate testing	**JC LD.04.05.01 EP1:** leadership offers clinical consultation
Analytic	Ensuring test methods and protocols comply with manufacturers’ requirements	**JC DC.02.01.01 EP1:** lab procedure requirements
	Ensuring test methodologies provide the quality of results needed for the population served	**CLIA §493.1445:** lab director responsibilities
	Recognize testing limitations, such as interference, and their potential impact on result interpretation	**CLIA §493.1251:** lab procedure requirements
Post-analytic	Ensuring reporting complies with manufacturers’ requirements	**JC DC.02.01.01 EP 1:** lab procedures requirements
	Ensuring appropriate follow-up testing is performed for certain testing scenarios	**CAP MIC.21835:** direct testing of positive blood culture broth for organism identification or antimicrobial susceptibility testing **CAP MIC.21950:** inconsistent antimicrobial susceptibility testing results **CAP MIC.22550:** CSF cultures back-up antigen testing **CAP MIC.65600:** mycobacterial cultures when performing *Mycobacterium tuberculosis* molecular testing on specimen **CAP IMM.41450:** HIV primary diagnosis requirements
	Clarity of results and informing providers of test performance	**CAP MIC.65630:** ensure clinical validity of molecular detection of antimicrobial resistance markers **CAP MIC.66100:** test method and clinical interpretation in reporting results **CLIA §493.1291:** test report requirements
	Consultation to support optimal result interpretation	**JC LD.04.05.01 EP1:** leadership offers clinical consultation
	Supporting AS and appropriate antimicrobial use	**JC MM.09.01.01:** hospital prioritizes AS **CAP MIC.21943** and **CAP MIC.42700:** selecting antibacterial and antifungal agents to report

^*a*^ FDA = Food and Drug Administration, LDT = laboratory-developed test, and CSF = cerebrospinal fluid.

### Upholding validated test requirements

Requests to test an off-label specimen: *Can a joint fluid specimen be tested using the rapid blood culture identification panel? There is substantial overlap between the pathogens of concern*.

*If a sample is sourced as a nasopharyngeal swab with a source comment that the collection is actually from the anterior nares, is it still acceptable to test?*

Requests to test an off-label indication: *Is it acceptable to employ a COVID test for asymptomatic patient screening if the indication for use only states testing patients with respiratory symptoms?*

Request for the laboratory to release non-validated results: *Can the laboratory report a cycle threshold value for a positive respiratory syncytial virus (RSV) result from a qualitative test?*

Laboratories are responsible for ensuring that all clinical testing adheres to the clinical and/or analytic validation studies performed prior to offering the test for patient care. DxS involves upholding these requirements in circumstances when testing an unvalidated or off-label specimen type is requested or discovered. DxS may further involve developing long-term interventions that shift testing practices toward validated solutions. **Joint Commission standard DC.02.01.01, EP 1** emphasizes the need for laboratory procedures that ensure all pre-analytic, analytic, and post-analytic steps comply with the manufacturer’s instructions (33). All stages of testing must be performed in accordance with the manufacturer’s instructions for a Food and Drug Administration (FDA)-approved or -cleared test (**CLIA § 493.1252**) (34). Instructions for use include acceptable specimen types and collection devices, clinical indications for testing (e.g., presenting syndromes and patient populations), how to handle and perform the test, and how to report results, including whether to report qualitative (positive or negative) or quantitative (i.e., viral loads; viral load surrogates such as cycle threshold values) results. If any clinical claims are not included in the manufacturer’s instructions of an FDA-approved or -cleared test, **CAP standard COM.40625** stipulates that laboratories must independently validate off-label clinical claims prior to testing (35).

When performing a laboratory-developed test (LDT), that is not FDA-approved, validation studies are required prior to testing and must account for pre-analytic variables and risks to test performance. The **CAP standard COM.40640** requires that the laboratory validate the performance characteristics of all specimen types for LDTs (35). The **CAP standard MOL.31015** provides guidelines for the extent of validation required when additional or multiple specimen types are validated as an LDT for molecular testing (36). Additionally, since regulatory requirements for LDTs are evolving, laboratories should continue to determine if test registration, pre-market submission, or other LDT-related regulations apply (37).

To comply with these requirements, the laboratory is responsible for educating providers about which patients are approved or validated for testing including variables such as clinical indication, age, gender, or specimen type. This also means that the laboratory is responsible for rejecting non-approved or non-validated specimens. The most effective approach to complying with these accreditation requirements is to provide tools that help providers select an appropriate test and collect the correct specimen up front, mitigating the need to reject specimens after collection. Such tools may include an informative laboratory test directory, direct communication to provider groups regarding a commonly misused test, careful electronic order build, and decision support tools to offer the appropriate test for a given population or dissuade inappropriate test selection for patients without an appropriate indication (9, 38). Working closely with stakeholders to understand the clinical and workflow needs that lead to unvalidated requests not only opens communication and mutual understanding but can also prompt the identification of alternative solutions for validated testing (e.g., sendout testing or bringing in a new test).

### Supporting optimal test quality and utilization

Multiple diagnostic approaches are often available for the detection of the same pathogen, but not all assays or methods are sufficient or equal. Performing a given test may be compliant with the manufacturer’s instructions or laboratory validation but does not guarantee it is the best test for a specific patient. It is the laboratory’s role, through DxS, to guide optimal quality and appropriate testing by quality-driven interventions strategically implemented throughout the phases of testing, as required by accreditation regulations and exemplified through the following scenarios.

Pre-analytic interventions: *Why are repeat specimen collections recommended for ova and parasite evaluation but rejected for routine stool culture or C. difficile testing?*

*Is anaerobic culture on a bronchoalveolar lavage specimen routinely acceptable?*

The interpretative guidelines of **CLIA standard §493.1240** define the pre-analytic phase to include test requests, specimen submission, specimen handling, and specimen referral (39). The laboratory is required to support pre-analytic quality by defining, communicating, and enforcing specimen acceptability or rejection. For example, the laboratory ensures quality and appropriateness of testing when defining the appropriate number of stool specimens and timing of collection when submitted for routine bacterial testing in accordance with the **CAP standard MIC.22440**. This standard suggests that laboratories work with clinicians to develop relevant policies, such as accepting no more than two specimens per patient for stool cultures without consultation about the limited diagnostic yield. DxS is also central to **CAP standard MIC.22675** which requires laboratories to define acceptable specimen sources for anaerobic culture to ensure interpretable results and **MIC.65600** in which laboratories must perform or recommend a mycobacterial culture when *M. tuberculosis* molecular testing is ordered (40).

Analytic and post-analytic interventions: *Should the laboratory repeatedly test the same sample instead of recommending recollection when a molecular test is indeterminate due to internal control failure? Recommending recollection may delay results*.

The laboratory is also responsible for minimizing the occurrence of issues with analytic quality and ensuring clear communication of test performance. Every test has technical limitations. The laboratory is required to account for these in procedures and recognize the impact of test limitations, such as interfering substances (**CLIA standard §493.1251**) and is required to provide test performance information that may impact test result interpretation upon request (**CLIA standard §493.1291**). This reporting guidance, including test limitations and guidance for interpretation, contributes to post-analytic quality. For example, when evidence of inhibition is noted during the analytic phase of a nucleic acid amplification test (NAAT; e.g., shift in or lack of the internal control signal), the laboratory is responsible for ensuring that a potentially erroneous result is not used to guide clinical care (**CAP standards MIC.65230, MIC.65250**) (40). When a rapid method is used for antimicrobial susceptibility testing, the laboratory is required to determine the extent of confirmatory testing to ensure quality results (**CAP MIC.21835**). DxS can underscore quality considerations during the pre-analytic and post-analytic phases to minimize suboptimal test requests and mitigate inaccurate result interpretation for patients likely to be most impacted by analytic limitations. Appropriate post-analytic interventions could include a call from the laboratory to the provider offering to place the specimen in a re-draw queue or a reporting comment explaining the reason for the indeterminate or invalid test result and recommending recollection, if clinically indicated. Lastly, laboratories are required to include a summary of the test method to inform clinical interpretation (**CAP MIC.66100**).

Algorithm-based testing: *HIV NAATs are highly sensitive and specific. Should a diagnostic HIV-1 NAAT be used as the first-line test for routine patient screening to obtain a faster but accurate result?*

Laboratory stewardship is critical when multiple tests may be required to optimally diagnose a single etiologic agent of infection. Furthermore, due to differences in performance characteristics, the type and order of the tests matter. Two scenarios are common. First, screening tests with high sensitivity but lower specificity may require confirmatory testing, such as a positive HIV-1/2 antibody rapid screen requiring follow-up with a fourth- or fifth-generation test (HIV-1/2 antibody and antigen) in accordance with the Centers for Disease Control and Prevention (CDC) diagnostic algorithm. Notably, HIV-1 qualitative NAAT is recommended in certain cases, and its recommended use depends on the results of the antibody/antigen and antibody differentiation tests (41). In this example, the **CAP standard IMM.41450** requires that the laboratory follows public health recommendations for HIV primary diagnosis, including either reflexing or providing clear guidance of required next steps to providers, such as supplemental or confirmatory testing, as appropriate (42). In a second common scenario, a rapid, low-sensitivity test may provide a fast diagnosis if positive but would require follow-up testing with a higher-sensitivity test if negative. Laboratory DxS can support effective and accurate diagnoses in both scenarios. For example, **the CAP standard MIC.22550** requires that if antigen-based methods are used for rapid detection of bacterial pathogens in cerebrospinal fluid, follow-up cultures are performed (40). To aid in navigating testing algorithms, laboratories often implement automatic reflexive testing or employ result comments recommending the use of a specific follow-up test or consultation requested by the appropriate specialist (microbiologist, pathologist, and infectious disease). These post-analytic interventions round out a comprehensive stewardship approach.

### DxS via clinical consultation

*Why is a clinical consultation required to order next-generation sequencing tests at many institutions? Should it be required at our institution?*

The **Joint Commission standard LD.04.05.01**, **EP1** requires that laboratory leadership offer consultative services (33). The requirement to have a Clinical Consultant to inform test ordering and result interpretation is also stated in **CAP standard GEN.53650** for laboratories performing moderate or high-complexity testing (43). Furthermore, laboratories are required to have procedures that specifically address the appropriateness of testing (for example, **Joint Commission standard QSA.15.01.01, EP2** regarding molecular testing) (33). **CLIA standard §493.1445** dictates that it is the responsibility of the CLIA laboratory director to ensure that the test methods employed by the laboratory provide “the quality of results required for patient care” (34). Thus, laboratory directors and delegated clinical consultants are required to provide their expertise to ensure appropriate test selection and result quality for the patients served by their laboratory. Recently, metagenomic next-generation sequencing for pathogen detection has become increasingly considered for clinical use and highlights an important application for clinical consultation paired with order approval requirements. Requiring clinical microbiologist or infectious disease consultations prior to ordering aims to increase the pre-test probability of detecting a true infection while mitigating negative outcomes due to misinterpretation of clinically insignificant positive or negative results (5). Additionally, clinical consultation with an infectious disease expert to aid the interpretation of results has been recommended (44).

Laboratory-driven requests or requirements for test consultation may be variably received by clinical providers depending on the content and context of the consultation request. In scenarios in which the consultation is mandated for order approval or to recommend an alternative test, DxS intervention processes may be most effective when developed through consensus by key stakeholders.

### Supporting AS

*Should we include pre-analytic entry question-response restrictions for the C. difficile NAAT order? The questions slow down the ordering workflow and can delay patient care.*

*Should the laboratory report all antibiotic agents for a bacterial isolate recovered from a urine culture in case the patient has a systemic infection?*

In March 2020, the Centers for Medicare and Medicaid Services (CMS) implemented the “Medicare and Medicaid Programs: Regulatory Provisions to Promote Program Efficiency, Transparency, and Burden Reduction Final Rule,” requiring acute-care hospitals to maintain AS programs. The U.S. federal law further requires that critical access hospitals must have an AS program to meet federally mandated goals (**42 CFR §485.640)** (45). This is similarly enforced by hospital accreditation agencies, such as by the Joint Commission (**Joint Commission standard MM.09.01.01**) (46). The U.S. CDC defines the core elements of a hospital AS program (47). The first core element is hospital leadership commitment, of which the microbiology laboratory is a key supporter for guiding appropriate antimicrobial selection and use. AS programs and DxS efforts share a common goal of improving patient care while reducing unnecessary treatment. Successful programs will prevent overutilization of diagnostic tests, thereby minimizing the risk of false detection of microorganisms, reducing unnecessary treatments that may lead to further spread of antimicrobial resistance, and lowering healthcare-associated costs.

Optimizing *C. difficile* testing is a prime example of DxS and AS working hand-in-hand. The laboratory may restrict testing based on pre-approved criteria (e.g., unexplained and new onset ≥3 watery stools in the past 24 hours, patient not receiving laxatives) that are determined in collaboration with AS and infectious disease experts and reject formed stool. These DxS interventions simultaneously ensure pre-analytic quality, avoid wasting financial resources by testing a low-yield patient without proper indications, mitigate post-analytic misinterpretation of positive test results that are more consistent with colonization rather than infection, and aid AS by avoiding unnecessary antimicrobial treatment (48).

The laboratory also supports AS by guiding providers to select only organism and body-site-appropriate antimicrobial agents and nudging them toward the narrowest ones possible through selective drug reporting. **CAP standards MIC.21943** and **MIC.42700** require that only antibacterial and antifungal agents appropriate to organism and body site are routinely reported (40). For guidance to implement these requirements, the reader may refer to Table 1A–1J in Clinical & Laboratory Standards Institute (CLSI) document M100 on designing and reporting antimicrobial panels (49). Importantly, the development of selective and cascade antimicrobial reporting protocols must be done in close collaboration with the AS team and other stakeholders, such as infectious disease specialists (**CAP standard MIC.21943**) (40). In addition to choosing which antimicrobials to report, the laboratory must identify and investigate unusual or inconsistent antimicrobial susceptibility results whether they are phenotypic or molecular detection of resistance markers (**MIC.21950 and MIC.65630**). Appropriate selection, testing, and reporting of antimicrobials highlight the role of diligent stewardship in all phases of antimicrobial susceptibility testing.

### Reporting compliance

*Should patient test results be immediately transmitted to the patient portal or set to a delayed transfer schedule to enable the caregiver to review the results first and provide appropriate patient consultation?*

Laboratory directors are responsible for ensuring that patient test result reporting adheres to the Cures Act. This means that results must be released to the patient portal as soon as they are available. Results should be clear and understandable to a patient, as results may be received before a physician is able to add their interpretation to the results or after-hours when a physician is not available to comment (50). Exceptions to information release blocking are rare but may be made for certain scenarios, especially when they could cause harm to the patient (51). When preparing or revising result reporting, collaboration with and receiving input from patient-facing providers regarding high-profile or complicated tests can inform how to best present scientifically accurate, but patient-friendly reports. The increased transparency of laboratory results to patients additionally reinforces the importance of DxS in optimizing appropriate test utilization.

### Optimizing reimbursement for clinically indicated testing

*Should a broad molecular respiratory panel be restricted to certain diagnoses and pre-approved clinical indications? Patients often specifically request this test.*

The role of DxS in optimizing test reimbursement is twofold: (i) discourage testing with a low likelihood of impacting clinical management, especially if alternative testing is sufficient and more likely to be reimbursed, (ii) ensure that opportunities for reimbursement of clinically indicated testing are maximized by understanding the clinical needs and common scenarios for testing for the population served. To maintain compliance with billing, maximize test reimbursement, and avoid unnecessary patient costs, laboratories must be aware of and adhere to payer requirements and regulations around reporting. For example, some Medicare Administrative Contractors (MACs) require additional documentation and/or limit reimbursement for broad multiplex molecular panels or when certain types of tests are ordered on the same day for a single patient. Some panels, such as urinary tract infection molecular panels, and repeat (duplicate) testing may not be covered by insurance at all. Palmetto MolDx, an example MAC, requires clinical validity and clinical utility test documentation, including published evidence of utility, from laboratories as part of determining coverage (52). Each potential combination of assays used (e.g., if influenza PCR, *Bordetella* PCR, SARS-CoV-2 PCR are performed from a single sample to evaluate a pediatric patient with a cough and fever) must be submitted for consideration as multiple independent standalone assays can be considered a panel.

While a test may still be performed if clinically necessary, even when not covered, such scenarios can lead to high patient or institutional charges. DxS, in these cases, can help ensure the test is truly clinically indicated, is the most appropriate test, and is likely to impact patient management. In these cases, every effort should be made to make the provider and the patient aware of the potential costs associated with the test. Examples of DxS interventions to reduce non-covered tests without substantial clinical necessity could include provider-directed education, electronic order restrictions, institutional algorithms that limit broad testing and promote narrow testing, and automatic order cancellations with notification for duplicate tests.

### Selecting reference services

*There is a novel test offered by a CLIA-certified specialty laboratory. Should the laboratory offer this test?*

*There is no CLIA-certified laboratory testing option for the requested service, but a research laboratory is willing to perform the test. Should the laboratory send samples to the research lab for patient testing?*

Clinically indicated tests that are not performed onsite or within a hospital system must be referred to an external laboratory. It is the responsibility of the laboratory, specifically the laboratory director and delegated consultants (**CLIA §493.1445**), to select reference laboratories that provide CLIA-compliant, high-quality, and clinically indicated tests, and there is a role for multidisciplinary DxS in determining which reference services are optimal and appropriate for the patients served and clinical situations encountered. The laboratory must ensure that the pre-analytic steps done onsite are performed in accordance with a selected reference laboratory’s requirements (**CLIA §493.1242**), and the pre-analytic requirements match the needs of the patients and providers, such as sample stability and transport acceptability (34). The **CAP standard GEN.41350** and **Joint Commission standard LD.04.03.09 EP1** further emphasize the responsibilities of the laboratory in selecting and evaluating referral laboratories, and **Joint Commission standard LD 04.03.09 EP10** requires that reference and contracted laboratories comply with clinical laboratory federal regulations (33, 43). Such an evaluation includes analytic and post-analytic considerations such as test methodology and performance, reporting thresholds or criteria, and how results are presented in the reports. For referral testing for patients under research protocol, **CAP standard GEN.41350** requires the laboratory to ensure that, if such testing is used for patient management decisions, the referral laboratory is CLIA-certified or else appropriately licensed to perform clinical testing according to CMS (43).

Selection of reference services should be done with the input and agreement of non-laboratory stakeholders. Laboratory expertise can help non-laboratory clinical providers understand the requirements an external lab must meet to be allowed to perform patient testing and to provide consultation regarding performance, clinical appropriateness, and any quality concerns or potential limitations of external lab tests before referring patient samples. Another key variable is the expected turnaround time for send out testing and availability of couriers and laboratory informatics interfacing to facilitate timely testing and reporting. A common diagnostic approach to ensuring oversight of referral services is for a laboratory to develop a list of pre-approved laboratories and corresponding tests, while any request outside the pre-approved list requires microbiologist or pathologist approval.

## DXS EXPERIENCE REQIREMENTS FOR TRAINEES

For clinical laboratories that train residents and fellows in programs accredited by the Accreditation Council for Graduate Medical Education (ACGME), many ACGME Common Program Requirements include elements that are directly addressed by trainee participation in DxS activities. Such requirements include education, training, and participation in patient safety improvement and quality improvement processes, including active involvement in interprofessional teams. Programs must ensure trainees become competent in systems-based practices and demonstrate healthcare cost awareness and value-based care (53, 54). Fellows training in the American Society for Microbiology (ASM) Subcommittee on Postgraduate Educational Programs (CPEP) fellowships in medical and public health microbiology are required to actively participate in all aspects of laboratory management and must demonstrate an understanding of “appropriate test utilization (e.g., cost effectiveness, operations, and clinical benefits) and identification of stakeholders to include in assessments of appropriate use” (55). Fellows must also participate in quality assurance and quality improvement activities and demonstrate the skills to be responsive to client needs and ensure quality patient care. Furthermore, trainees who interact routinely with the laboratory (e.g., infectious disease fellows, pharmacists) also benefit from experience with DxS, in addition to their training in microbiology and antimicrobial stewardship. These areas of laboratory training are integral to the principles of DxS.

## STRATEGIES TO IMPLEMENT EFFECTIVE DIAGNOSTIC STEWARDSHIP

Laboratories employ various methods to meet the standards of high-quality testing, ensure regulatory compliance, and guide best testing and result interpretation, all of which are components of DxS. While some analytic quality assurance activities are managed independently by laboratorians, most pre-analytic and post-analytic, and many analytic DxS activities are best managed through interdisciplinary participation. Developing and organizing DxS activities into an effective program requires institutional support, with many systems finding success through DxS committees. Efforts to pursue the implementation of DxS require systems-based thinking and can be modeled after AS programs. To be most effective, such programs require (i) a governance with interdisciplinary team members, (ii) interventions to improve patient care, (iii) data extraction and monitoring and (iv) data review with the outcome of implementing quality improvement tactics (56). Many stakeholders will be involved during the process, often including, but not limited to: infectious disease physicians, healthcare providers across multiple specialties, quality and safety officers, information technology specialists, public health authorities, hospital administrators, medical technologists/laboratory scientists, and sometimes the patients themselves.

A variety of interventions can be employed for DxS and, often, more than one intervention may be required to maximize the impact for a given stewardship project. For example, provider education is a broad approach that is essential to accomplishing almost all DxS goals. This can come in the form of directed verbal education, memos, carefully crafted advisories (or “best practice alerts”), reporting comments, and more. Similarly, clinical consultation may be employed at any phase of testing to promote a desired action or interpretation while mitigating potential, unintentional negative outcomes of testing (57). Upholding specimen requirements is another broad, but highly impactful intervention in which all members of the laboratory help support DxS. Interventions that leverage tools in the electronic medical record are increasingly used (cumulatively referred to as “computerized clinical decision support”) such as question-response logic-based order guidance, carefully crafted test names, advisories linked to test orders, test results, or other patient conditions indicated in the medical record, and others (58). Post-analytically, the presentation of results and reporting comments can guide the interpretation of results, patient management, or direct the provider toward the best next step, whether that is additional testing or how to seek consultation from an expert (microbiologist, infectious disease, AS, or other) (1).

Implementing any stewardship intervention does not come without risks, so this process should be carefully coordinated with stakeholder involvement. One of the biggest potential concerns raised by altering testing practices through DxS interventions is delayed or missed diagnoses (59). While there is a lack of published examples demonstrating such outcomes, limiting testing combined with fears of missed diagnoses could hypothetically negatively impact patient outcome or drive physicians to empirically treat patients, undermining AS efforts, if not implemented optimally. Additionally, any DxS interventions employed without interdisciplinary support or supportive education may result in provider dissatisfaction and reduce the effectiveness of the program. Careful planning, collaboration, and constant monitoring will help to mitigate risks associated with implementing a diagnostic stewardship program. Each program should review the institution’s current diagnostic practices, including test utilization patterns, workflow processes, and resource availability. Areas of inefficiency, overutilization, or diagnostic errors and examining the causes of misutilization may inform targeted interventions to pursue (60). To gather support for a given stewardship intervention, data-driven approaches and effective communication around the rationale, benefits, and evidence are essential. Presenting relevant data and evidence demonstrating the impact of diagnostic errors, inappropriate test utilization, or antimicrobial resistance on patient outcomes, healthcare costs, and AS efforts is an important aspect of these conversations. Real-world examples or case studies can help illustrate the need for improved diagnostic practices. Engaging key opinion leaders, offering educational resources and training sessions, addressing concerns and misconceptions, and sharing success stories from other healthcare institutions can further bolster support. Pilot projects or phased approaches to demonstrate the feasibility and effectiveness of a program on a smaller scale may help build consensus. It is also important to measure performance and evaluate the effectiveness of an intervention after implementation (60).

## SYSTEMS THINKING WITH DXS

**Fig 1:**
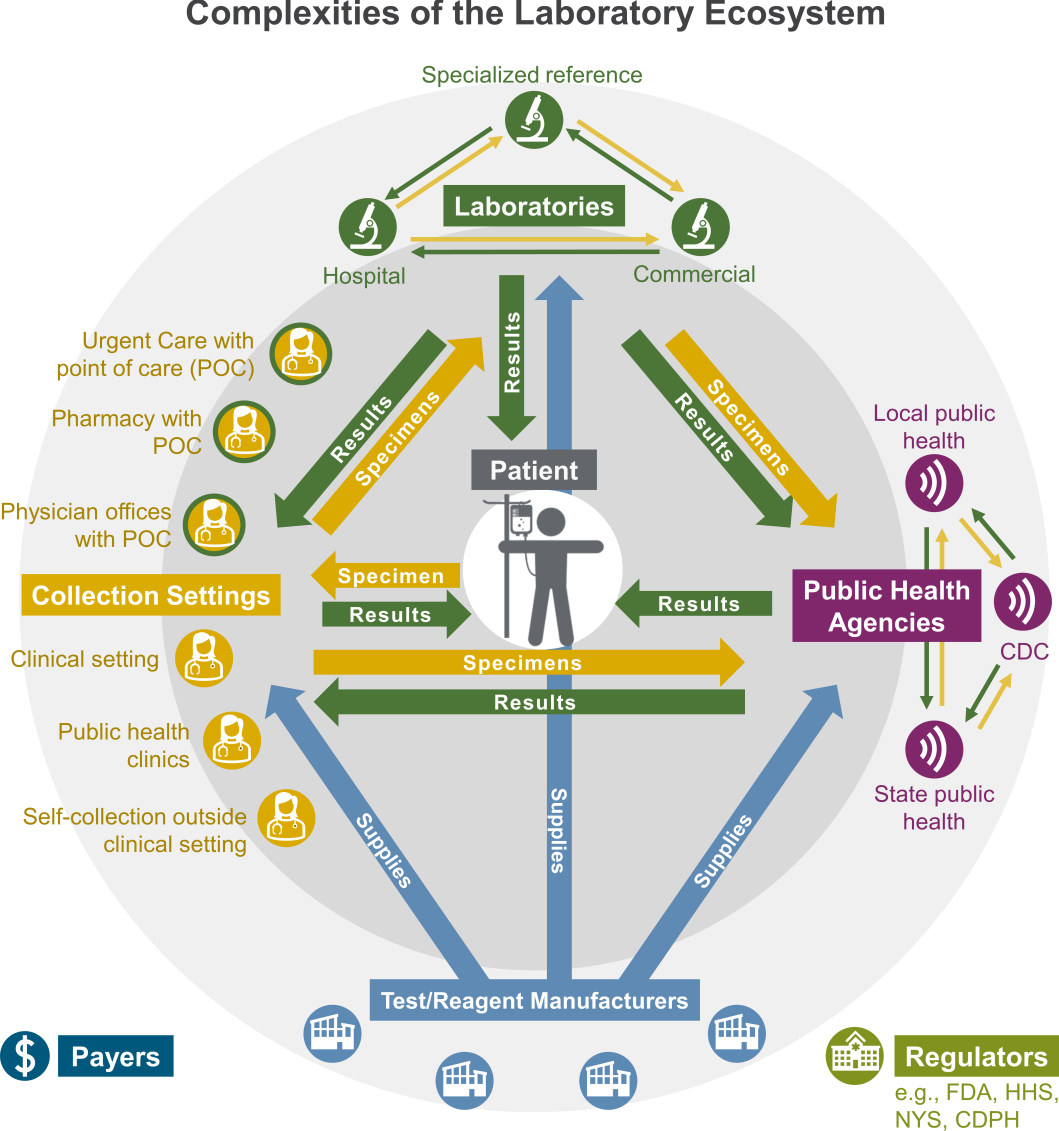
The laboratory ecosystem. The laboratory ecosystem is composed of a complex network of collection sites, testing sites, and test/reagent manufacturers, which must all work in coordination to best serve our patients, at the center. Specimens are collected in clinical and non-clinical settings (yellow circles) and must efficiently be transported to testing sites (yellow arrows). Test results may be obtained at the collection site when testing is performed at the point of care (yellow circles with green outlines) or at clinically focused laboratories (green circles) and public health laboratories (purple circles). Test results must be transmitted back to care providers, often by way of intermediary labs, and the patients from all testing sites (green arrows). To perform the testing, collection devices, reagents, and tests are supplied by the manufacturers (blue circles) and must be consistently and efficiently available at all stages of testing (blue arrows). Regulators and payers also play an essential role in the laboratory ecosystem’s ability to offer high-quality, affordable care.

Within an organization, effective DxS requires a culture of laboratory involvement and initiatives that are driven at a system level (56). Nationally, the network of clinical and public health laboratories in the United States is made up of many interconnected stakeholders, as depicted in Fig. 1. While the patient remains at the center of testing, the nation’s network of laboratories often functions in a semi-coordinated, yet siloed fashion. Thus, improving DxS requires a broader systems view of the clinical laboratory ecosystem. In the United States, patient testing starts at the front-line of care in a hospital, clinic or point-of-care facility, which can provide different levels of services depending on the size and structure of the health system. Large medical centers, which often offer broader patient care with subspecialty expertise and robust administrative structures, are the most likely to have organizational support for a DxS program. Conversely, private physician practices and smaller hospitals may have limited access to extensive in-house testing and potentially limited resources for an active DxS program. Organizations with limited in-house testing menus often rely on reference laboratories to provide a substantial proportion of patient testing, whereas organizations with more extensive in-house test menus may still rely on reference laboratory services for specialized and/or lower-volume testing. However, fee-for-service referral organizations are not responsible for or incentivized to perform significant DxS beyond regulatory requirements. Thus, to promote optimal testing and DxS interventions relating to testing sent out from their facilities, institutions may directly monitor send-outs using strategies such as creating a laboratory test formulary (61). Despite such efforts, DxS for referred testing presents unique challenges that require collaboration across the laboratory ecosystem to be most effective.

Clinical laboratories also refer specimens to their local and state public health laboratories who work in coordination with the U.S. CDC to perform testing for organisms and conditions of public health concern. The network of U.S. public health laboratories includes state and local facilities, who are subspecialized to track and respond to public health threats. The U.S. public health laboratory network also includes a subset of specialized facilities that comprise the nation’s Laboratory Response Network (LRN), which has the capability to respond to high-priority biological agents and local outbreaks using standardized protocols (62).

Clinical laboratories and public health laboratories work closely with government agencies, such as the U.S. FDA and CMS, to ensure quality testing is readily available. These government agencies provide federal oversight and guidance on policies related to human testing. CMS has regulatory oversight of the clinical laboratories through the CLIA and FDA has oversight of *in vitro* diagnostic testing through FDA approval or clearance and the Emergency Use Authorization (EUA) process, which was implemented during SARS-CoV-2 and Mpox outbreak responses (63). Commercial manufacturers and suppliers of diagnostic solutions are also vital to this laboratory ecosystem and work closely with both the U.S. FDA and clinical and public health laboratories to indirectly provide patient care.

Professional societies such as the CAP, ASM, American Clinical Laboratory Association, Association of Public Health Laboratories, American Society for Clinical Pathology, the Infectious Disease Society of America, and others often represent their membership to support guidelines and advocate for funding and legislation that impact the laboratory community. Evidence-based and best-practice guidelines from national society experts often provide the basis and clinical justification for DxS projects.

A modernized laboratory ecosystem in the age of big data must be viewed as a strategic asset. Annually in the United States, roughly 14 billion laboratory tests are ordered and performed in any of the almost 260,000 CLIA-licensed laboratories (64). Thus, the laboratory is not only vital for providing quality test results but also functions as a data command center that can continue to be leveraged to make better evidence-based population health decisions. By bringing together a collection of key individuals with expertise across the ecosystem of the clinical and public health laboratory system, new innovative approaches and solutions can be generated. However, to move the needle in laboratory medicine on DxS will require systems thinking beyond local institutional policies.

## CONCLUSIONS

DxS for infectious diseases is an all-encompassing process to promote the highest quality and value of clinical testing to enable accurate and efficient diagnoses and treatment. While the term “diagnostic stewardship” has gained new momentum and focus over the past couple decades, many of the core elements and interventions of DxS are not new, as exemplified throughout this document. However, the ever-evolving infectious disease *in vitro* diagnostic market, pressures on clinical resources including reimbursement, and regulatory oversight rules require continued focus and interdisciplinary collaborations to optimize test utilization and subsequent impacts on clinical care. Additionally, new advances and opportunities in microbiology testing, such as testing methods or result interpretation driven by artificial intelligence-based algorithms and an increasing market for at-home and direct-to-consumer testing, present future arenas for microbiology expert-driven DxS.
